# Identifying the content of home-based health behaviour change interventions for frail older people: a systematic review protocol

**DOI:** 10.1186/s13643-015-0138-8

**Published:** 2015-11-04

**Authors:** Ana Jovicic, Benjamin Gardner, Celia Belk, Kalpa Kharicha, Steve Iliffe, Jill Manthorpe, Claire Goodman, Vari Drennan, Kate Walters

**Affiliations:** Department of Primary Care and Population Health, University College London, Royal Free Hospital, Rowland Hill Street, London, NW3 2PF UK; Department of Psychology, Institute of Psychiatry, Psychology and Neuroscience (IoPPN), King’s College London, London, UK; UCL Centre for Behaviour Change, University College London, London, UK; Social Care Workforce Research Unit, King’s College London, London, UK; Centre for Research in Primary and Community Care, University of Hertfordshire, Hertfordshire, UK; Centre for Health and Social Care Research, St George’s, University of London, London, UK

**Keywords:** Protocol, Older people, Frailty, Intervention, Behaviour change

## Abstract

**Background:**

Meeting the needs of the growing number of older people is a challenge for health and social care services. Home-based interventions aiming to modify health-related behaviours of frail older people have the potential to improve functioning and well-being. Previous reviews have focused on whether such interventions are effective, rather than what might make them effective. Recent advances in behavioural science make possible the identification of potential ‘active ingredients’ of effective interventions, such as component behaviour change techniques (BCTs), and intended intervention functions (IFs; e.g. to educate, to impart skills). This paper reports a protocol for a systematic review that seeks to (a) identify health behaviour change interventions for older frail people, (b) describe the content of these interventions, and (c) explore links between intervention content and effectiveness. The protocol is reported in accordance with Preferred Reporting Items for Systematic Reviews and Meta-Analyses Protocols (PRISMA-P) 2015 guidelines.

**Methods/design:**

Studies will be identified through a systematic search of 15 electronic databases, supplemented by citation tracking. Studies will be retained for review where they report randomised controlled trials focusing on home-based health promotion delivered by a health professional for frail older people in community settings, written in English, and either published from 1980 onwards, or, for registered trials only, unpublished but completed with results obtainable from authors. Interventions will be coded for their content (BCTs, IFs) and for evidence of effectiveness (outcome data relating to behavioural and health outcomes). Analyses will describe characteristics of all interventions. Interventions for which effectiveness data are available will be categorised into those showing evidence of effectiveness versus those showing no such evidence. The potential for each intervention characteristic to contribute to change in behaviour or health outcomes will be estimated by calculating the percentage of all interventions featuring those characteristics that have shown effectiveness.

**Discussion:**

Results will reveal the strategies that have been drawn on within home-based interventions to modify the health behaviours of frail older people, and highlight those more associated with positive changes in behaviour and health. Findings from this review will provide a useful basis for understanding, developing, and implementing behaviour change interventions in this field.

**Systematic review registration:**

PROSPERO CRD42014010370

**Electronic supplementary material:**

The online version of this article (doi:10.1186/s13643-015-0138-8) contains supplementary material, which is available to authorized users.

## Background

In the UK, the number of people aged over 85 years is projected to rise from 1.4 million in 2012 to 5 million by 2050 [1]. Providing services to meet their needs represents a great challenge for health and social care services because functional limitations [2], falls [3], and multi-morbidity [4] are common among very old people. Frailty has been defined as a clinically identifiable state of increased vulnerability, resulting from ageing-related declines in reserve and functioning [5, 6]. This has been operationalised as meeting certain ‘phenotypic’ criteria, including low energy or exhaustion, low physical activity, slow walking speed, low grip strength, and unintentional weight loss [7]. Among people aged 65 or over, prevalence of frailty has been estimated at around 11 %, while a further 42 % are estimated to have mild frailty or ‘pre-frailty’ [8]. Prevalence rates rise with increasing age and multi-morbidities [8]. Frailty has been associated with increased risk of disability, admission to hospital or moves to care homes, and death [5, 7, 9, 10]. Frailty is not inevitable. Ageing is not a linear process of progressive decline [11], and it is possible to address some accumulating deficits at stages when they are still tractable.

Home-based health promotion shows potential to reduce functional declines in frail and pre-frail populations [12, 13]. Such interventions often include attempts to modify the health-related behaviour of older adults, such as increasing dietary quality or levels of physical activity [14]. Developing new behaviour change interventions, or refining existing ones, can however be costly and time-consuming, and so, evidence is needed to identify the intervention components most likely to change behaviour and thereby improve health [15]. Recent advances in behavioural science have generated methods for furthering understanding of the ‘active ingredients’ of behaviour change interventions. A taxonomy is available of 93 discrete behaviour change techniques (the ‘BCT Taxonomy v1’ [16]), which may be used to change any behaviour (e.g. providing information on health consequences, providing social support, using prompts or reminders). A framework for understanding and changing behaviour (the ‘Behaviour Change Wheel’ [17]) outlines nine discrete functions that any intervention may serve (e.g. persuasion, education, skills training). Intervention functions (IFs) represent ‘broad categories of means by which an intervention may change behaviour’ ([18] p. 109), and BCTs the irreducible components that serve to deliver such functions. The technique taxonomy and IF list can be applied retrospectively to identify within published intervention descriptions which methods have been drawn on to change behaviour [19–21]. This is important for several reasons. First, the techniques employed in behaviour change interventions are typically poorly described [22], and imposing the terminology of the BCT Taxonomy offers a means to standardise description of intervention methods, so aiding replication of effective interventions. Second, the taxonomy also links BCTs to theory, and so, documenting the techniques used in previous interventions can reveal implicit assumptions regarding the causes of behaviour and how it might be changed [23]. For example, an intervention centred on providing information on the importance of physical activity in older adulthood assumes that physical inactivity can be attributed to insufficient knowledge [24]. This can be especially useful to understand interventions that have not explicitly drawn on specific theories of behaviour or behaviour change. Third, comparing the techniques and functions of effective and ineffective interventions may reveal the techniques that are most promising for changing a focal behaviour [21, 25]. Previous reviews have focused on the extent to which home-based health promotion interventions are effective for older adults [22], but not what may make them effective.

This review aims to describe the components that have featured in previous home-based health behaviour change studies for older people, and assess the extent to which these components are associated with intervention effectiveness. This will inform the development of home-based health promotion interventions for frail and pre-frail older people.

### Objectives

This review has the following objectives. In accordance with Preferred Reporting Items for Systematic Reviews and Meta-Analyses Protocols (PRISMA-P) guidance [26], these are set out with reference to PICO (participants, interventions, comparators, outcome) criteria:

1. Identify health behaviour change *interventions* for frail older people (*participants*) delivered by health professionals within the home environment, compared against no treatment or usual-care treatments (*comparators*), reporting effects on behavioural or health-related *outcomes*
2. Describe intervention content: specifically, the BCTs and intervention functions used and theoretical bases for interventions
3. Explore relationships between intervention components and intervention effectiveness, as defined as positive between-group changes in favour of the intervention condition on at least one behavioural or health outcome

## Methods/design

### Design

This systematic review is based on PRISMA reporting guidelines [27], supplemented by the application of structures for coding intervention techniques and functions (See Additional file 1 for PRISMA-P checklist). This review is one of two concurrent reviews of evidence around home-based health promotion for older people. The current review assesses the BCTs and functions used within home-based health promotion interventions that have been evaluated among older people using randomised controlled trial (RCT) designs. The partner review will focus on effectiveness of interventions with older people with mild frailty in particular, and will include all types of study design. Both reviews are registered under the same entry in the PROSPERO database (CRD42014010370).

### Eligibility criteria

#### Participants

Interventions will be entered into the review where they have been trialled among *community-dwelling adults aged 65 years and over with frailty or at risk of frailty*. Interventions trialled only on older adults living in residential or nursing care home settings (long-term care facilities) or hospital inpatients will be excluded. We will deem populations to be frail or at risk of frailty where they have been assessed as having frailty or pre-frailty using a validated frailty measure, or, where frailty was not directly measured, considered to be at risk of frailty, identified by being at risk of hospitalisation, having functional or mobility difficulties, or aged over 75 years with multiple morbidities. Interventions trialled among those aged 50 years or above will be eligible if the mean age of the sample was 65 years or above. Where an intervention has been trialled on participants with a single health condition and includes an intervention specific to that population and/or to addressing symptoms related to that condition (e.g. cognition or behavioural symptoms in people with dementia or rehabilitation interventions after a stroke), the trial will be excluded.

#### Intervention

The review will focus on *interventions that aim to modify health-related behaviours of older adults and have been delivered in person within the participant’s home by health professionals*, *but do not require specialist professional expertise for delivery*. We define behaviours to be ‘health-related’ where they may reasonably be expected to impact on mental or physical health, regardless of whether undertaken (or not undertaken) for health reasons [28]. Interventions that feature only non-behavioural components—such as changes in prescribed medications (a pharmacological intervention), signposting to other services (organisational intervention), or that include behaviour change components focusing solely on the behaviour of health professionals or others responsible for care provision for older adults—will be excluded. Interventions delivered exclusively or primarily outside of the participant’s place of residence, such as in healthcare settings or group activities in community venues, will also be excluded. If an intervention is delivered in multiple settings including the participant’s place of residence, it will be included only where quantitative outcome data are available relating exclusively to the home-based component. Interventions not delivered face-to-face nor in-person (e.g. telephone counselling), or that depend on the skills of a specialist (e.g. exercise interventions that require delivery by a physiotherapist or postural stability instructor or nutritional interventions that require dietician expertise), will be excluded. The latter exclusion criterion has been set so that the findings are applicable to inform the development of interventions suitable for delivery by health professionals without these specialist skills (e.g. practice or community nurses) or trained health advisors from a lay or non-professional background.

#### Comparators

Only studies that employ a randomised controlled trial design to *compare at least one intervention against no treatment*, or *a usual-care treatment*, will be included. Where multiple interventions are reported in a single trial, only data pertaining to those interventions that meet the remaining criteria will be included in the review. We will record how papers describe their controls in case of variability in usual-care across studies.

#### Outcomes

Only *primary quantitative outcome data relating to health-related behaviours*, *or health outcomes relevant to older people with frailty*, will be included. Behavioural outcomes include initiation of health behaviour change (i.e. uptake of positive health behaviour or reduction or cessation of health-risk behaviour) or maintenance of existing positive health behaviour. We define ‘positive health behaviour’ as ‘any activity that may help to prevent disease, detect disease and disability at an early stage, promote and enhance health, or protect from risk of injury’ ([28], p. 18; e.g. physical activity). Conversely, ‘health-risk behaviour’ refers to any activity with the potential to cause disease or hinder its prevention, cause or hinder the detection of disease and disability at an early stage, diminish health, or cause injury or hinder protection from risk of injury (e.g. smoking). ‘Health behaviour’ is used as an umbrella term that encompasses both positive health behaviour and health-risk behaviour. Health outcomes deemed relevant to older people with frailty include worsening frailty, well-being, quality of life, disability, functioning (activities of daily living), mobility, hospitalisation, admission to care homes, negative affect (anxiety, depression, mood), and cognitive functioning. Outcomes referring to satisfaction with the intervention, or any other service, will not be eligible.

#### Study type

Papers will be entered into the review where they are *available in English in full-text*, *report primary quantitative data from randomised controlled trial designs*, *and are either published within peer-reviewed sources between 1980 and 2014*, *or*, *for registered trials only*, *unpublished but completed and with results obtainable from the authors*. Where results from the same trial are reported across multiple sources, information from these sources will be pooled and treated as a single trial for the purpose of analysis.

### Information sources and search strategy

#### Information sources

Eligible studies will be identified via two sources. First, searches will be conducted using the following health and medical electronic databases: MEDLINE; MEDLINE in Process & Other Non-Indexed Citations; EMBASE; SCOPUS; Science Citation index Expanded (SCIE); Cochrane Database of Systematic Reviews (CDSR); Cochrane Central Register of Controlled Trials (CCRCT); Cochrane Effective Practice and Organisation of Care Group (EPOC); PsycINFO; Health Technology Assessment (HTA) database; National Health Service Economic Evaluation Database (NHS EED); Health Economics Evaluations Database (HEED); Cumulative Index to Nursing and Allied Health Literature (CINAHL); Evidence for Policy and Practice Information Centre Register of Health Promotion and Public Health Research (BiblioMap); and Health Development Agency Register (Health Promis). As-yet-unpublished studies will be identified through searches in the HTA database, the UK Clinical Research Network Portfolio Database, and ClinicalTrials.gov.

Second, backwards, forwards, and lateral citation-tracking searches will be undertaken of relevant papers. Papers will be eligible for citation tracking where they are either (a) systematic reviews that are retained following screening of abstracts against eligibility criteria or (b) intervention trial evaluations deemed eligible for inclusion in the review following full-text screening. Citation tracking will be undertaken by manually searching reference lists (backwards citation tracking), conducting cited-reference searches on Web of Science, Google Scholar and Scopus (forwards citation tracking), and using the ‘related articles’ function on PubMed and Web of Science (lateral citation tracking).

#### Database search strategy

The search strategy will use key words derived from our population, intervention, comparator, and outcome and study type inclusion criteria, as detailed above (see Additional file 2 for example search strategy). The search terms have been developed iteratively over several team meetings with the wider research team and trialled on online databases to ensure that they retrieve as many relevant results as possible. Preliminary searching will begin with a strategy based on keyword/index (Medical Subject Headings (MeSH)) terms. The initial database searches and title screening will be conducted by a researcher (AJ, a health psychologist).

#### Eligibility screening

Following de-duplication of the study database, all records will be independently screened for eligibility by two researchers, a health psychologist (AJ) and a general practitioner (CB). Eligibility screening will occur in two stages: first, abstracts will be screened to remove records that are clearly ineligible, and second, full-text copies of remaining records will be obtained and screened. Any uncertainty or disagreements between researchers will be recorded and resolved through discussions with senior members of the research team, who have experience in older adulthood and frailty (KK, KW), clinical general practice (KW, SI), and health psychology and behaviour change (BG).

### Data extraction

References will be managed using EndNote databases, with different databases used to represent the data corpus at each of the searching and screening stages. Behaviour change intervention descriptions tend to be poorly specified [29]. To maximise the likelihood of accurately and comprehensively coding intervention content for review purposes, corresponding authors of all papers included in the final dataset will be emailed and asked to provide all available additional information relating to the intervention (e.g. study protocol, intervention manual, related published journal papers).

A standardised data extraction form will be developed, and Microsoft Excel files will be used for entering and storing extracted data. All sources will be coded for study characteristics (methodological characteristics, sample characteristics, study quality, outcome data) and intervention characteristics (behaviour[s] targeted, BCTs, intervention functions, explicit theory-basis, delivery, fidelity). Data extraction will be undertaken by AJ and a second, independent coder. A senior research team member with experience of systematic reviewing, and applying behaviour change coding technologies to evidence synthesis (BG), will independently extract data from at least 20 % of studies. The primary data extractor (AJ) has been fully trained in applying the BCT Taxonomy v1 to code intervention reports via a free online training programme provided by the authors of the BCT Taxonomy (http://www.bct-taxonomy.com/). Inter-rater reliability will be assessed via percentage agreement and kappa (κ) statistics, calculated using a Microsoft Excel macro. Reliability statistics will be generated for all study and sample characteristics combined, and each intervention characteristic (e.g. behaviour[s] targeted, BCTs, intervention functions) in turn. Disagreements will be addressed via discussions between coders, with deference to a third coder (KW) where these are not resolved. If, after an initial second-coding exercise, at least one kappa value is less than ‘substantial’ (i.e. ĸ < 0.60 [30]), the second-coder will code an additional batch of 5–10 % of sources and calculate new reliability statistics. This process, aimed at identifying and eliminating systematic coding errors, will continue until all kappa values are substantial (ĸ ≥ 0.60).

#### Study characteristics

For descriptive purposes, each study will be coded for the following methodological characteristics: country in which study undertaken, setting (e.g. exclusively within the home or home and other setting), study design, length of all follow-ups, number of arms and interventions, and risk of bias (at study-level). Risk of bias will be assessed using the Cochrane Collaboration tool for RCTs [31], which includes items relevant to the research topic and design. Trials will be classified into low, high, or unclear risk of bias. All records (100 %) will be second-coded for bias risk by CB, with any disagreements resolved through deferral to a third coder (BG). All eligible studies will be entered into the evidence synthesis, regardless of possible bias.

#### Sample characteristics

The following characteristics will be extracted for eligible intervention and control treatment arms: participant eligibility criteria, baseline and follow-up sample size, demographics (age range, gender, ethnicity), and prevalence of health conditions in the sample.

#### Intervention characteristics

Where possible, the following information will be extracted from each eligible intervention described within each source. We define an ‘intervention’ as any treatment, other than usual care, which is designed to change participants’ behaviour or their mental or physical health. Where an intervention includes behavioural components aimed at both older people and others (e.g. health professionals, case managers), the following information will be extracted only for those components aimed at older people.

##### Behaviours targeted

Health promotion interventions can differ in the precise behaviours they recommend as a means to improving health (e.g. dietary intake, physical activity, medication adherence), and so the behaviour or behaviours targeted in the intervention will be recorded. Behaviours will be coded only where explicitly mentioned in the intervention description.

##### Behaviour change techniques

BCTs will be coded using the standardised coding manual of the BCT Taxonomy v1, a reliable 93-item coding frame [16]. Each of the 93 BCTs will be coded as either present or absent. BCTs will be coded only where there is unambiguous evidence of their presence in the protocol. Where the intervention content delivered differs across intervention recipients, BCTs will be coded where they clearly have been delivered to at least one recipient. Frequency of BCT delivery will not be coded.

##### Intervention functions

Interventions will be coded according to whether they are designed to perform one or more of nine possible intervention functions: education, persuasion, incentivisation, coercion, training, restriction, environmental restructuring, modelling, and enablement [17]. Functions will be coded using descriptions and examples taken from the function list provided by Michie et al. ([17], p. 7).

##### Theory-basis

A 19-item checklist is available which sets out the ways in which behaviour change theories can be drawn on when designing and evaluating interventions (e.g. to identify the determinants of the target behaviour, to select recipients, to select or tailor change techniques [32]). Yet, generally, intervention descriptions rarely draw on theory, and those that do typically specify only that one or more theories *have* been used, rather than *how* they have been used [32, 33]. In anticipation of a low level of theory use, interventions will initially be coded using an adaptation of one of Michie and Prestwich’s [32] items, namely whether a specific and named theory of behaviour or behaviour change has been explicitly mentioned in the abstract, introduction, or method. If named theories are found to be present in at least 25 % of papers, Michie and Prestwich’s coding frame will be applied comprehensively.

##### Delivery and fidelity

Who delivers the intervention, and whether records are kept of how faithful the intervention as delivered was to the delivery protocol, will be coded for descriptive purposes.

#### Outcome data

We will extract quantitative outcome data pertaining to all available behavioural (e.g. exercise levels, smoking), health and well-being indices (e.g. quality of life, activities of daily living), as measured at baseline and first reported follow-up point following the end of the intervention period.

Group means, effect sizes, confidence intervals, and *p* value data will be extracted. Exact *p* values will be coded where possible; otherwise, *p* value cut-offs (e.g. *p* > .05) will be extracted. Where baseline and follow-up data are unavailable and effects are reported (e.g. mean differences are provided in the absence of group means), only effect size, confidence interval, and *p* values will be extracted.

### Analysis

Two analyses will be run. The first will describe the dataset to identify the contents of previous interventions. The second will compare the content of those interventions for which sufficient outcome data are available to permit categorisation of the intervention as showing ‘evidence of effectiveness’ or ‘no evidence of effectiveness’.

#### Description of study dataset

All study, sample, and intervention characteristics, and a summary of study findings, will be presented for descriptive purposes. The implicit theoretical underpinnings of interventions will be inferred from the BCTs and intervention functions observed across the dataset.

#### Comparison of ‘effective’ vs ‘ineffective’ interventions

##### Clustering of outcomes

Relevant behavioural, health and functioning outcomes from across the dataset will first be inductively organised into thematic clusters. Outcome clusters will be mutually exclusive, so that no outcome may be classified into more than one cluster. Given the inductive nature of the thematic analysis, the final clusters cannot be anticipated, but we expect groupings such as positive health behaviour, health-risk behaviour, physical functioning, mental health, and so on.

##### Identifying effective interventions

For the purpose of analysis, an intervention will be deemed to ‘show evidence of effectiveness’ in bringing about change within an outcome cluster where it is associated with a statistically significant (*p* < .05) health-positive between-group change, at the first reported follow-up, in at least one outcome within that cluster. We define a ‘health-positive’ change as any observed change towards more engagement in positive health behaviours, less engagement in health-risk behaviours, or better health or functioning, experienced by an intervention group relative to control. Health-positive changes include maintenance of positive health behaviour, or maintenance of health or functioning, where a decline in any of these outcomes is experienced in the control, or a lesser decline in the intervention group than the control.

Interventions that report multiple outcomes relative to the same cluster will be deemed to ‘show evidence of effectiveness’ where there is health-positive change in at least one outcome within the cluster. Any intervention in which there is no health-positive change in relation to any outcomes within that cluster will be deemed to ‘show no evidence of effectiveness’ in relation to that cluster. Interventions for which no outcome data relating to a cluster are available will be excluded from analyses related to that cluster.

Where studies report multiple outcomes, each of which is categorised into a different outcome cluster, it is possible that the intervention may be deemed to show evidence of effectiveness in relation to one cluster and ‘show no evidence of effectiveness’ in relation to another. The definition of an intervention that shows evidence of effectiveness is purposefully broad, because the review aims to highlight interventions that show potential, however small, to improve behaviour, health, or well-being.

##### Associations between intervention characteristics and ‘evidence of effectiveness’

For each outcome cluster, the characteristics of interventions that show evidence versus those showing no evidence of effectiveness will be compared. For each coded intervention characteristic (i.e. behaviour targeted, BCT, IF), we will report the total number of interventions in which it has been used. We will also calculate a coefficient representing the number of interventions featuring the characteristic that show evidence of effectiveness, as a percentage of all interventions featuring that characteristic. We will deem intervention characteristics to ‘show evidence of promise’ where they have featured in at least four interventions in total, and have been used in more interventions that show evidence of effectiveness than those showing no evidence of effectiveness (i.e. coefficient >50 %). Intervention characteristics that yield a coefficient equal to or below 50 %—that is, that have featured in more interventions showing no evidence of effectiveness than those showing evidence of effectiveness, or an equal number of each—will be deemed to ‘show no evidence of promise’. We deem any characteristic that has featured in three or fewer interventions as having been used insufficiently to permit reliable classification of its promise.

## Discussion

This systematic review will describe the characteristics of behavioural home-based health promotion interventions for frail older people and those at risk of frailty, and identify the intervention components that show promise in yielding positive behaviour or health changes for this group. The coding process will allow for the systematic identification of common and replicable elements of promising interventions: what works, for whom, and why? While our analyses will point to intervention characteristics that show promise, these findings will be indicative, rather than conclusive. Syntheses of intervention components are constrained by the clarity of intervention descriptions, such that poorly reported components may not be identified, and those that are consistently well-reported may be over-represented in analyses [29]. While we will attempt to overcome this by contacting authors directly, response rates to such information requests are often low [25, 29]. Additionally, our analysis of BCTs and intervention functions focuses on their presence versus absence, not the frequency or intensity with which they have been used, nor how they have been delivered, by whom, and in what settings, because this information cannot be reliably observed from intervention reports [34]. Nonetheless, our findings will be useful in summarising, using standardised terminology, the behaviour change strategies that have been adopted in previous studies, and pointing future intervention designers to those strategies that have most frequently featured in promising interventions.

### Reporting and dissemination of findings

The review will be reported in accordance with PRISMA guidelines [27], and will be reported in a peer-reviewed journal and at relevant national and international conferences.

## Additional files

Additional file 1:
**Preferred Reporting Items for Systematic review and Meta-Analysis Protocols (PRISMA-P) 2015 checklist: recommended items to address in a systematic review protocol.** (DOCX 30 kb) Additional file 2:
**Sample search strategy, for Ovid Medline.** Table of search terms to enable our search to be replicated. (DOCX 12 kb)

## Abbreviations

BCT: behaviour change technique
HTA: Health Technology Assessment
IF: intervention function
MeSH: Medical Subject Headings
NHS: National Health Service
NIHR: National Institute for Health Research
PRISMA: Preferred Reporting Items for Systematic Reviews and Meta-Analyses
RCT: randomised controlled trial
