# Supplementary material for: Identifying the content of home-based health behaviour change interventions for frail older people: a systematic review protocol
Source: Syst Rev. 2015 Nov 4;4:151. doi: 10.1186/s13643-015-0138-8 (PMC4634580; doi:10.1186/s13643-015-0138-8)
Supplement: Additional file 2: — Sample search strategy, for Ovid Medline. Table of search terms to enable our search to be replicated. (DOCX 12 kb) [file 13643_2015_138_MOESM2_ESM.docx]

**Additional File 2: Sample search strategy, for Ovid Medline**

| **Step** | **Search term** |
| --- | --- |
| 1.  (Population) | (“old* age” OR “aging” OR “ageing” OR “old* adult*” OR “old* people” OR “elder*” OR “geriatric*” OR “senior*” OR “pensioner*” OR “over 65” OR “over sixty five” OR “over sixty-five” OR “65+” OR “veteran*” OR “frail*”).mp |
| 2.  (Intervention) | (“health promotion*” OR “behavio* chang*” OR “healthy aging” OR “healthy ageing” OR “health education” OR “intervention*” OR “lifestyle*” OR “wellbeing” OR “health campaign*” OR “health prevent*” OR “health protect*” OR “primary prevent*” OR “case manag*” OR “diet*” OR “nutrition” OR “healthy eating” OR “exercis*” OR “physical activit*” OR “alcohol” OR “smok*” OR “mood*” OR “depress*” OR “anxi*” OR “psycholog*” OR “cogniti*” OR “fall* prevent*” OR “polypharmacy” OR “prevent* hospital*”).mp |
| 3.  (Setting) | (“Home-based” OR “homebased” OR “house-based” OR “housebased” OR “community-dwelling” OR “community dwelling” OR “domiciliary” OR “outreach” OR “home”).mp |
| 4.  (Study type) | (“Trial” OR “randomi* control*” OR “RCT”).mp |
| 5. | 1 AND 2 AND 3 AND 4 |
| 6.  (Limits) | Limit 5 to (English language and full text and humans and yr=”1980-2014”) |
